# Digital Democracy and Emergency Preparedness: Engaging the Public in Public Health

**DOI:** 10.3389/ijph.2024.1608004

**Published:** 2024-12-20

**Authors:** Giovanni Spitale, Nikola Biller-Andorno, Federico Germani, Sonja Merten

**Affiliations:** ^1^ Institute of Biomedical Ethics and History of Medicine, University of Zurich, Zurich, Switzerland; ^2^ Swiss Tropical and Public Health Institute, Basel, Switzerland; ^3^ University of Basel, Basel, Switzerland

**Keywords:** emergency preparedness, public health, ethics, digital democracy, digital technology

## Introduction

The intersection of digital technology and democratic processes presents a transformative avenue for enhancing public health responses during health emergencies. This special issue, titled “Digital Democracy and Emergency Preparedness: Engaging the Public in Public Health,” explores how digital platforms and democratic engagement can work together to strengthen EP (emergency preparedness) and response mechanisms. The advent of digital technology has revolutionized the way information is disseminated and how communities engage with health authorities. From social media campaigns to mobile health apps, digital tools offer unprecedented opportunities for public participation in health-related decision-making processes [1–3]. This paradigm shift towards DD (digital democracy) in public health not only facilitates real-time communication and feedback but also empowers communities, ensuring their voices are heard and their needs addressed in times of crisis [4].

However, leveraging DD for public health is not without its challenges. Issues such as the digital divide, privacy concerns, fragmented governance structures, and the spread of misinformation pose significant hurdles to effective engagement [5–9]. Despite these challenges, the potential benefits of integrating digital tools with democratic practices in emergency preparedness are very promising [4].

This special issue explores critical aspects of public health during the COVID-19 pandemic, including communication strategies in nursing homes in Southern Switzerland Bernardi et al., the impact of social media overload on depressive symptoms among Chinese students Xie et al., the role of communicative behaviors and organizational reputation in shaping public health intentions Akbulut, public sentiment toward easing COVID-19 measures in China Xin et al., and the use of digital diary methodologies to capture real-time insights and amplify diverse voices during crises Kaiser-Grolimund et al.

## The Role of Digital Democracy in Public Health

DD offers a novel approach to confronting public health challenges, as digital platforms can be used to foster a more engaged and informed public [10]. This digital engagement is crucial for disseminating health-related information [3, 11, 12], for empowering communities to participate actively in health decision-making processes, and for building resilient health systems [13, 14].

Community empowerment in public health is one of the aims of the UN Sendai Framework for Disaster Risk Reduction [15]. DD facilitates this empowerment: incorporating it into public health initiatives aligns with the Sendai Framework’s call for a more inclusive and participatory approach to disaster risk management. This two-way exchange enhances the transparency and accountability of public health initiatives and ensures that EP and response strategies are grounded in community knowledge and experience [4, 13, 14]. By adopting DD tools, public health authorities can move beyond top-down communication, leading to a more dynamic, inclusive, and empowering approach to health governance.

## Emergency Preparedness and Public Engagement

Public engagement in the context of EP involves active participation, collaboration, and empowerment of communities to take charge of their health and safety. A truly resilient public health system is one that incorporates the public as a key stakeholder in this preparation [16–18]. Thus, engaging the public in EP involves educating communities, promoting a culture of preparedness. This ensures that emergency plans are reflective of and responsive to the needs and vulnerabilities of different communities, so that strategies are both effective and equitable [15]. Engaging the public helps to build trust between health authorities and communities. Trust facilitates the positive reception of accurate information and the negative reception of mis/disinformation, which undermines emergency response efforts [17, 19, 20]. Moreover, by involving local communities in the planning process of EP, authorities can harness local knowledge and insights, which are valuable for the creation of locally relevant EP measures [21–23]. DD plays a fundamental role in facilitating this engagement [3, 10, 12].

Effective EP thus requires a paradigm shift from a top-down approach to a collaborative model that values and incorporates public input [13]. This shift enhances the effectiveness of preparedness measures through the promotion of resilient communities. Arguably, engaging the public in EP is not just a strategic necessity, but also a moral obligation to ensure that communities are not merely passive recipients of monitoring or interventions, but active participants in safeguarding their health and wellbeing.

## Challenges and Ethical Considerations

The integration of DD into public health comes with its challenges and ethical considerations [9, 10]. These issues must be carefully considered to ensure that the benefits of digital engagement are realized without compromising individual rights or exacerbating existing inequalities. For example, the digital divide [24]: access to digital technologies presents significant disparities, and this can limit the effectiveness of DD initiatives. Privacy concerns represent another significant challenge. DD approaches in EP involve the collection, storage, and analysis of personal data. Without stringent data protection measures, there is a risk of privacy breaches, which can undermine public trust and deter participation [25, 26]. The rapid spread of infodemics can have profound consequences, undermining public health efforts and leading to confusion and panic. Combating infodemics while respecting freedom of expression requires a delicate balance [27–29]. Ethical considerations also extend to the design and implementation of DD initiatives, which should engage communities without reinforcing existing power imbalances. Participatory design processes can help ensure that digital tools are accessible, user-friendly, and culturally sensitive [13, 30, 31].

### Conclusion

The articles presented in this special issue highlight the importance of integrating DD into EP strategies. The convergence of digital tools and democratic engagement presents a powerful avenue to enhance public health responses to emergencies and to build more resilient communities, leveraging technology that facilitates communication and participation, using bottom-up and bi-directional approaches [4]. This special issue also underlines substantial gaps in our understanding and application of DD approaches in public health. It is evident that there is a strong need for continued research, innovation, and interdisciplinary collaboration. Thus, the advancements highlighted within these pages serve as a foundation for future work.

The journey towards integrating DD into public health EP is full of challenges. However, the potential rewards—more resilient communities, enhanced public engagement, and more effective emergency responses—underline the relevance of continuing these efforts.

## References

[1] Yoon S Goh H Devi Nadarajan G Sung S Teo I Lee J Perceptions of Mobile Health Apps and Features to Support Psychosocial Well-Being Among Frontline Health Care Workers Involved in the COVID-19 Pandemic Response: Qualitative Study. J Med Internet Res (2021) 23:e26282. 10.2196/26282 33979296 PMC8168635

[2] Sheehan M. Ethics and Policy: Dealing With Public Attitudes. Radiat Prot Dosimetry (2008) 129:295–8. 10.1093/rpd/ncn044 18593686

[3] ITU. Digital Tools and Strategies in COVID-19 Infodemic Response: Case Studies and Discussion (2021). Available from: https://www.itu.int:443/en/publications/ITU-D/Pages/publications.aspx (Accessed September 12, 2024).

[4] Spitale G Merten S Jafflin K Schwind B Kaiser-Grolimund A Biller-Andorno N. A Novel Risk and Crisis Communication Platform to Bridge the Gap between Policy Makers and the Public in the Context of the COVID-19 Crisis (PubliCo): Protocol for a Mixed Methods Study. JMIR Res Protoc (2021) 10(11):e33653. 10.2196/33653 34612823 PMC8562419

[5] Sekalala S Dagron S Forman L Meier BM. Analyzing the Human Rights Impact of Increased Digital Public Health Surveillance During the COVID-19 Crisis. Health Hum Rights J (2020) 22/2:7–20. PMC7762901 33390688

[6] Hsu Y-C Chen Y-L Wei H-N Yang Y-W Chen Y-H. Risk and Outbreak Communication: Lessons From Taiwan’s Experiences in the Post-SARS Era. Health Security (2017) 15(2):165–9. 10.1089/hs.2016.0111 28418746 PMC5404243

[7] Attademo G. International Efforts Against the Infodemic: Some Bioethical Reflections. Medicina e Morale (2022) 71(1):69–78. 10.4081/mem.2022.1200

[8] Spitale G Biller-Andorno N Germani F. Concerns Around Opposition to the Green Pass in Italy: Social Listening Analysis by Using a Mixed Methods Approach. J Med Internet Res (2022) 24(2):e34385. 10.2196/34385 35156930 PMC8852653

[9] Grande D Mitra N Luna Marti X Merchant R Asch D Dolan A Consumer Views on Using Digital Data for COVID-19 Control in the United States. JAMA Netw Open (2021) 4:e2110918. 10.1001/jamanetworkopen.2021.10918 34009347 PMC8134997

[10] Fagherazzi G Goetzinger C Rashid MA Aguayo GA Huiart L. Digital Health Strategies to Fight COVID-19 Worldwide: Challenges, Recommendations, and a Call for Papers. J Med Internet Res (2020) 22(6):e19284. 10.2196/19284 32501804 PMC7298971

[11] Wilhelm E Ballalai I Belanger M-E Benjamin P Bertrand-Ferrandis C Bezbaruah S Measuring the Burden of Infodemics: Summary of the Methods and Results of the Fifth WHO Infodemic Management Conference. JMIR Infodemiology (2023) 3(1):e44207. 10.2196/44207 37012998 PMC9989916

[12] Willaert T Van Eecke P Beuls K Steels L. Building Social Media Observatories for Monitoring Online Opinion Dynamics. Social Media Soc (2020) 6. 10.1177/2056305119898778

[13] Spitale G Germani F Biller-Andorno N. The PHERCC Matrix. An Ethical Framework for Planning, Governing, and Evaluating Risk and Crisis Communication in the Context of Public Health Emergencies. The Am J Bioeth (2023) 24:67–82. 10.1080/15265161.2023.2201191 37114888

[14] Biller-Andorno N Spitale G. 2022. “Addressing Volatile Ethical Issues of Covid-19 with the Core Five Enduring Values List for Health Care Professionals. NEJM Catalyst Innov Care Deliv. 10.1056/CAT.22.0108

[15] UN Office for Disaster Risk Reduction. Sendai Framework for Disaster Risk Reduction 2015-2030 (2015). Available from: https://www.undrr.org/publication/sendai-framework-disaster-risk-reduction-2015-2030 (Accessed September 12, 2024).

[16] Adhikari B Pell C Cheah PY. Community Engagement and Ethical Global Health Research. Glob Bioeth (2020) 31(1):1–12. 10.1080/11287462.2019.1703504 32002019 PMC6968663

[17] Adjekum A Blasimme A Vayena E. Elements of Trust in Digital Health Systems: Scoping Review. J Med Internet Res (2018) 20(12):e11254. 10.2196/11254 30545807 PMC6315261

[18] Bishop BJ Vicary DA Browne AL Guard N. Public Policy, Participation and the Third Position: The Implication of Engaging Communities on Their Own Terms. Am J Community Psychol (2009) 43:111–21. 10.1007/s10464-008-9214-8 19184410

[19] Lee CW. Who Is Community Engagement for? The Endless Loop of Democratic Transparency. In: American Behavioral Scientist. SAGE Publications Inc (2020). 10.1177/0002764220945358

[20] Solomon MZ Gusmano MK Maschke KJ. The Ethical Imperative and Moral Challenges of Engaging Patients and the Public with Evidence. Health Aff (2016) 35:583–9. Project HOPE. 10.1377/hlthaff.2015.1392 27044955

[21] Jao I Kombe F Mwalukore S Bull S Parker M Kamuya D Involving Research Stakeholders in Developing Policy on Sharing Public Health Research Data in Kenya: Views on Fair Process for Informed Consent, Access Oversight, and Community Engagement. In: Journal of Empirical Research on Human Research Ethics. SAGE Publications Inc (2015). 10.1177/1556264615592385 PMC4548475 26297748

[22] Wilson P Helen Mavhandu-Mudzusi A. Working in Partnership with Communities to Improve Health and Research Outcomes. Comparisons and Commonalities between the UK and South Africa. Prim Health Care Res Development (2019) 20:e129. NLM (Medline). 10.1017/S1463423619000677 PMC6739449 31500680

[23] Pinto RM Spector AY Rahman R. Nurturing Practitioner-Researcher Partnerships to Improve Adoption and Delivery of Research-Based Social and Public Health Services Worldwide. Int J Environ Res Public Health MDPI AG (2019) 16:862. 10.3390/ijerph16050862 PMC6427324 30857292

[24] Dijk J. The Digital Divide. John Wiley & Sons (2020).

[25] WHO. WHO Guidelines on Ethical Issues in Public Health Surveillance. WHO (2017). Available from: https://www.who.int/publications/i/item/who-guidelines-on-ethical-issues-in-public-health-surveillance (Accessed September 12, 2024).

[26] Samuel G Ahmed W Kara H Jessop C Quinton S Sanger S. Is It Time to Re-Evaluate the Ethics Governance of Social Media Research? In: Journal of Empirical Research on Human Research Ethics. SAGE Publications Inc (2018). 10.1177/1556264618793773 30141738

[27] Boender TS Schneider PH Houareau C Wehrli S Purnat TD Ishizumi A Establishing Infodemic Management in Germany: A Framework for Social Listening and Integrated Analysis to Report Infodemic Insights at the National Public Health Institute. JMIR preprints (2023) 3:e43646. 10.2196/43646 PMC10273031 37261891

[28] Machiri S Purnat T Nguyen T Ho C Ballalai I Biller-Andorno N An Ethics Framework for Social Listening and Infodemic Management. The Eur J Public Health (2023) 33(Suppl. 2):ckad160–661. 10.1093/eurpub/ckad160.661

[29] Purnat TD Nguyen T Briand S editors Managing Infodemics in the 21st Century: Addressing New Public Health Challenges in the Information Ecosystem. Cham: Springer International Publishing (2023). 10.1007/978-3-031-27789-4 39556674

[30] Gurung G Tuladhar S. Fostering Good Governance at Peripheral Public Health Facilities: An Experience from Nepal. In: Rural and Remote Health. James Cook University (2013). Available from: https://www.scopus.com/inward/record.uri?eid=2-s2.0-84877890785&partnerID=40&md5=9264e6c7124872e132af7686b1ebc6ff (Accessed September 12, 2024). 23528140

[31] Molster C Maxwell S Youngs L Kyne G Hope F Dawkins H Blueprint for a Deliberative Public Forum on Biobanking Policy: Were Theoretical Principles Achievable in Practice? Health Expect (2013) 16:211–24. 10.1111/j.1369-7625.2011.00701.x 21645188 PMC5060653

